# Comparison of the prevalence of kidney disease by proteinuria and decreased estimated glomerular filtration rate determined using three creatinine-based equations among patients admitted on medical wards of Masaka Regional Referral Hospital in Uganda: a prospective study

**DOI:** 10.1186/s12882-022-02865-w

**Published:** 2022-07-07

**Authors:** SSenabulya F. Ronny, Nankabirwa I. Joaniter, Kalyesubula Robert, Wandera Bonnie, Kirenga Bruce, Kayima James, Ocama Posiano, Bagasha Peace

**Affiliations:** 1 Department of Medicine, Makerere University College of Health Sciences, P.O.BOX 7062, Kampala, Uganda; 2 Infectious Disease Research Collaboration, Kampala, Uganda; 3 Makerere NCD Training Program, Makerere Lung Institute, Kampala, Uganda; 4 Mulago National Referral Hospital, Kampala, Uganda; 5 Uganda Heart Institute, Mulago Hospital, Kampala, Uganda

**Keywords:** Kidney disease, Comparison of prevalence of kidney disease, Estimated glomerular filtration rate equations

## Abstract

**Background:**

Despite estimated glomerular filtration rate (eGFR) being the best marker for kidney function, there are no studies in sub-Saharan Africa comparing the performance of various equations used to determine eGFR. We compared prevalence of kidney disease determined by proteinuria of ≥  + 1 and or kidney disease improving global outcomes (KDIGO) eGFR criteria of < 60 ml/minute/1.73m^2^ determined using three creatinine-based equations among patients admitted on medical ward of Masaka Regional Referral Hospital.

**Methods:**

This was a prospective study conducted among adult patients admitted on medical wards between September 2020 to March 2021. Spot urine samples were collected to assess for proteinuria and blood samples were collected to assess serum creatinine levels. Kidney disease was defined as proteinuria of ≥ 1 + on spot urine dipstick and or KDIGO eGFR criteria of < 60 ml/minute/1.73m^2^. Estimated glomerular filtration rate was calculated using three creatinine-based equations: a) Full Age Spectrum equation (FAS), b) chronic kidney disease-Epidemiology collaboration (CKD-EPI) 2021 equation, c) CKD EPI 2009 (without and with race factor) equation. CKD was determined after followed up at 90 days post enrollment to determine the chronicity of proteinuria of ≥  + 1 and or KDIGO eGFR criteria of < 60mls /minute/1.73m^2^. We also compared prevalence of CKD determined by KDIGO eGFR criteria of < 60mls /minute/1.73m^2^ vs age adapted eGFR threshold criteria for defining CKD.

**Results:**

Among the 357 patients enrolled in the study, KDIGO eGFR criteria of < 60mls / minute determined using FAS and CKD-EPI 2009 without race factor equations and or proteinuria of ≥  + 1 showed the highest overall prevalence of kidney disease at 27.2%.

Prevalence of confirmed CKD at 90 days was highest with proteinuria ≥  + 1 and or KDIGO eGFR criteria of < 60mls/min determined using CKD EPI 2009 without race factor Equation (15.1%).

**Conclusions:**

Use of KDIGO eGFR criteria of < 60mls / minute /1.73m^2^ using FAS and CKD-EPI 2009 without race equations identifies the largest number of patients with CKD. Health care systems in sub-Saharan Africa should calculate eGFR using FAS equations or CKD-EPI 2009 without race equations during basic screening and management protocols.

**Supplementary Information:**

The online version contains supplementary material available at 10.1186/s12882-022-02865-w.

## Background

The burden of kidney disease is rising worldwide, with estimates in 2017 at 29.5% compared to the 19.7% in 2007 [1]. Mild abnormalities in kidney structure and function are associated with increased risk for complications & mortality [2]. Kidney disease may present as acute kidney injury (AKI), acute kidney disease and disorders (AKD) or as chronic kidney disease (CKD). Kidney failure refers to eGFR of < 15mls/minute/1.73m^2^ of AKI,AKD or CKD requiring treatment by dialysis or transplantation [3, 4]. Therefore, early screening and identification of patients at risk or with kidney disease allows prompt management of patients before development of kidney failure related complications and mortality.

In Africa there are no studies comparing of CKD prevalence with the various estimated glomerular filtration rate (eGFR) equations. However in Uganda, a few studies evaluating kidney function have used different eGFR equations in different settings including use of Cockcroft Gault formula and Modification of Diet in Renal Disease (MDRD) in outpatient clinic setting [5], use of Cockcroft Gault formula in a community-based setting [6] and use of chronic kidney disease-Epidemiology collaboration (CKD-EPI) with race factor equation among special populations at high risk of kidney disease [7].

These studies have shown a generally high burden of kidney disease in these settings. However, no studies have compared the estimated magnitude of kidney disease by comparison prevalence with different equations in any Ugandan health care facility. Appreciating which equation identifies the largest number of patients with kidney disease or CKD at regional referral hospitals is important as these facilities are first point and sometimes the only point of care for most patients in the country. This study compared the prevalence of kidney disease by proteinuria ≥  + 1 and or KDIGO eGFR criteria of < 60mls/minute/1.73m^2^ determined using three eGFR equations among patients admitted on medical wards of Masaka Regional Referral Hospital (RRH).

## Methods

### Study design and setting

This was a prospective study conducted among adult patients admitted on the medical wards of Masaka RRH. This is the largest government hospital in Masaka district, which is located in south central Uganda. The hospital provides both preventive and curative services to over two million people. This hospital has 330 bed capacity with the medical wards using up over 21% of the hospital beds. Medical wards admit patients with various medical conditions including but not limited to anemia, liver cirrhosis, heart failure, malaria, diabetes mellitus and hypertensive disorders. The hospital has no specialized nephrology clinic. However, it runs a daily general medical outpatient clinic, hypertension and diabetes mellitus clinics at least once a week.

### Study population and procedures

**Fig. 1:**
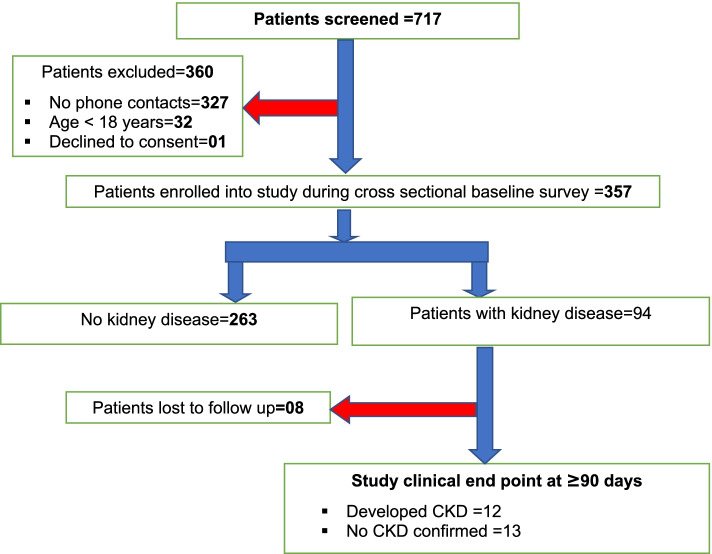
Patient flow chart from enrollment to end of the study: Flow chart shows that 717 patients were screen, 360 excluded basing on exclusion criteria

All adult patients admitted to the medical wards of Masaka RRH between September 2020 and November 2020 were consecutively screened for eligibility to join the study. Patients were enrolled if: 1) were aged 18 years and above; 2) were admitted on medical wards for at least 24 h; 3) provided written informed consent. Patients were excluded if they had no phone contacts to allow follow-up. Details are shown by study patient flow chart in Fig. 1.

A detailed questionnaire was administered to all enrolled patients by study personnel. The questionnaire captured information on demographics and risk factors for kidney disease. A venous blood sample and a midstream spot urine sample were collected from all patients using aseptic techniques. Approximately 4mls of venous blood was drawn from each patient into a syringe then placed into a red top vacutainer to assess for serum creatinine. The eGFR were calculated using the chronic kidney disease-Epidemiology collaboration (CKD-EPI) 2021 equation which was used for identifying the patients with kidney disease in the field for sub-sequent follow-up. Given that CKD-EPI 2021 equation has not been validated in Africa, at analysis, eGFR has also been estimated using two additional creatinine-based equations: a) Full Age Spectrum (FAS) equation with specific Q values for African people and b) CKD EPI 2009 (without and with race factor) equation [8–12], however, findings from these estimates were not used for participant management. All patients were provided with a sterile urine container and educated briefly about provision of 20 mls of early morning mid-stream urine to assess for proteinuria.

Follow up phone call was conducted for all patients meeting the definition of kidney disease, 90-days following the baseline assessment. During the phone call, we established if patient was either still alive or had deceased but without establishing cause of death. Kidney disease was defined as proteinuria of ≥ 1 + and or kidney disease improving global outcome (KDIGO) eGFR criteria of < 60 mls/minute/1.73m^2^ [2]. Patients who were still alive were requested to return to the medical outpatient clinic for a repeat venous blood sample collection to measure the post 90-days serum creatinine and spot urine dipstick testing for proteinuria.

Outcome of interest as patients returned for clinic visit after 90 days was either CKD or no confirmed CKD. Clinical end point of the study was: 1) no confirmed CKD, 2) developed CKD. Lost to follow up (LTFU) was defined as going 2 months without attending a scheduled study visit. Chronic kidney disease was defined as ≥ 90 days of KDIGO eGFR criteria of < 60mls /min /1.73m^2^ and or proteinuria of ≥  + 1. Interval censoring strategy was used to determine the study outcomes.

Prevalence of CKD was also compared between the KDIGO eGFR criteria of < 60 ml/min/1.73m^2^ and with the age-adapted eGFR threshold definitions for CKD [13].

### Laboratory evaluations

The venous blood collected was centrifuged within one hour of collection to obtain serum. Sample analysis for serum creatinine was conducted at Masaka RRH laboratory using Jaffe method traceable to an isotope dilution mass spectrometry [14]. SCr was measured using COBAS machine (C311) manufactured by (Roche diagnostics, North America) with a standard calibration reference range of 66-106umol/L. Urine samples were tested within 30 min of collection for proteinuria using a 10-parameter dipstick (Urinspect strips) manufactured by Artron laboratories.

### Data management and statistical analysis

All data was entered into Microsoft office excel database, then exported to STATA version 13 software package for analysis. Continuous and discrete variables were summarized into medians with interquartile ranges (IQR). Categorical variables were summarized into frequencies and percentages. Proportions and bivariate analysis were used to identify risk factors associated with kidney disease by deriving percentages, odds ratios (OR), confidence intervals (CI) and *P* value for the respective relationships. *P*-value of ≤ 0.05 was considered statistically significant.

The prevalence of kidney disease was calculated as total number of patients with proteinuria of ≥ 1 + and or decreased eGFR of < 60mls/min/1.73m^2^ as calculated by each eGFR equation divided by the total number of patients enrolled. Proportion of patients who developed chronic kidney disease was calculated as number of patients with ≥ 90 days of decreased eGFR of < 60mls/min/1.73m^2^ as calculated by each equation and or proteinuria divided by total number of patients with kidney disease at baseline.

Prevalence of kidney disease at baseline determined by age adapted eGFR threshold definition was calculated as number of patients having age adapted eGFR definition of CKD divided by total number of patients enrolled.

Proportion of patients who developed CKD was calculated as number of patients with age-adapted eGFR threshold definitions of CKD at ≥ 90 days divided by number of patients followed up having kidney disease by age-adapted eGFR threshold definition of CKD at baseline screening.

## Results

### Characteristics of the study population

Between September and November 2020, a total of 717 patients were screened for eligibility to join the study, of which 357 (49.8%) were enrolled. Reason for exclusion of the 360 potential patients included lack of a phone contact to allow follow-up 327/360 (90.8%), age < 18 years 32 (8.9%), and 01 (0.3%) refused to consent to participate in the study. The median age of the 357 patients enrolled was 47 (IQR 32–63) years, males constituted 52.9%, and majority of had no or only primary education (75.9%). Details of baseline characteristic are presented in Table 1 below.

**Table 1:** Characteristics of the study patients

Characteristic	Proportions (%) *N* = 357
**Age categories: Median (IQR)**	
18 – 35	28 (20–31)
36 – 59	47 (40–52)
60 years and above	73 (66–80)
**Sex**	
Female	168 (47.1)
Male	189 (52.9)
**Tribe**	
Baganda	241 (67.5)
Banyakitara	52 (14.6)
Others^a^	64 (17.98)
**Occupation**	
Civil/NGO servant	20 (5.6)
Operate business	73 (20.5)
Peasant farmer	177 (49.6)
Unemployed	49 (13.7)
Others^b^	38 (10.6)
**Religion**	
Born again and SDA	33 (9.24)
Catholic	202 (56.6)
Moslem	78 (21.9)
Protestant	44 (12.3)
**Level of Education**	
No education	55 (15.4)
Primary	216 (60.5)
Secondary	65 (18.2)
Tertiary	21 (5.9)
**Marital status**	
Divorced/separated	76 (21.3)
Married	148 (41.4)
Single	58 (16.3)
Widow/widower	75 (21.0)

**Others**.^a^: Acholi, Iteso, Basoga

**Other**.^b^: Carpenter, Builder and manual laborer

### Comparison of prevalence of kidney disease

**Fig. 2:**
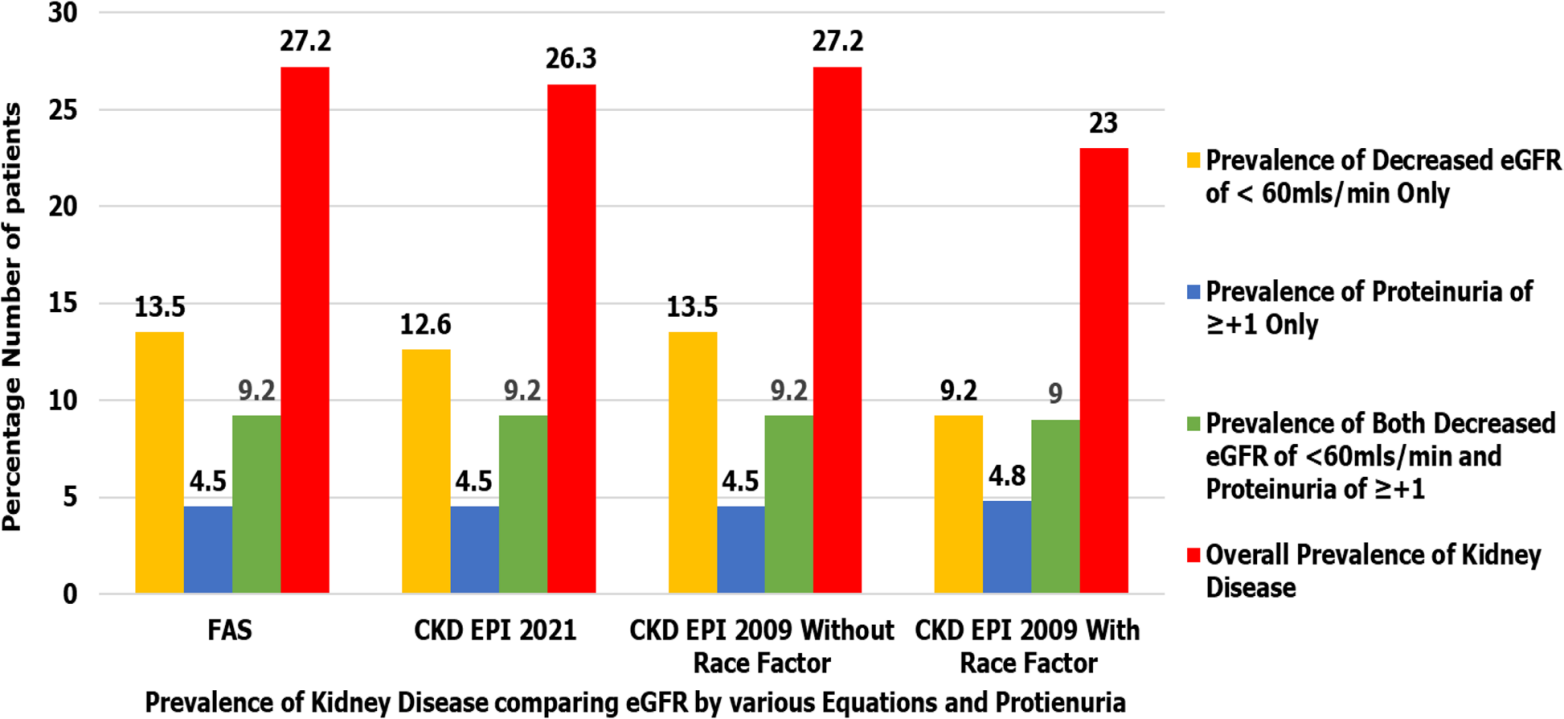
Comparison of prevalence of kidney disease basing on proteinuria ≥  + 1 and or KDIGO eGFR criteria of < 60 ml/min/1.73m^2^ calculated by various eGFR creatinine-based equations. Bar chart shows that among the 357 patients enrolled in the study, both FAS and CKD EPI 2009 without race factor equations and or proteinuria of ≥  + 1 revealed the highest overall prevalence of kidney disease at 27.2% while CKD EPI 2009 with race factor and or proteinuria of ≥  + 1 showed the lowest overall prevalence of kidney disease at 23%. *N* = Number of patients enrolled in the study

**Fig. 3:**
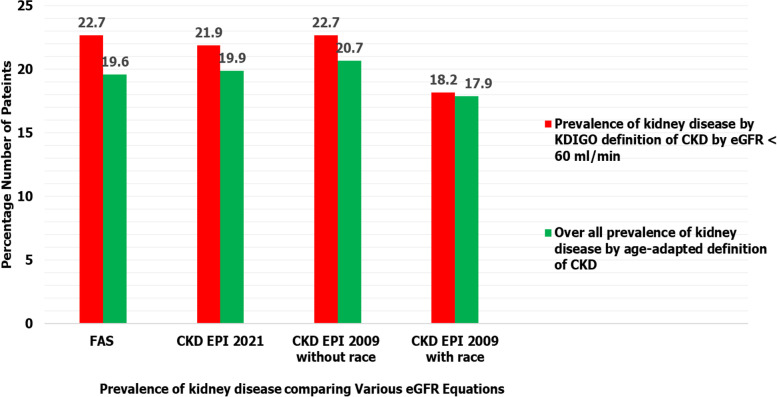
Comparison of prevalence of kidney disease using KDIGO eGFR criteria (< 60 ml/min/1.73m^2^) for defining CKD vs age adapted eGFR thresholds for CKD definition. Bar chart shows that KDIGO eGFR criteria of < 60 ml/min/1.73m^2^ for defining CKD identifies 0.3–3% slightly more patients with kidney disease than age adapted eGFR thresholds for CKD definition while using all eGFR serum creatinine-based equations. *N* = Number of patients enrolled in the study

Out of 357 patients enrolled, the overall prevalence of kidney disease was compared using proteinuria ≥  + 1 and or KDIGO eGFR criteria of < 60 ml/min/1.73m^2^ calculated by various eGFR creatinine-based equations as shown in details by Fig. 2 below. Prevalence of Kidney disease was also compared between KDIGO eGFR criteria of < 60 ml/min/1.73m^2^ vs age-adapted eGFR thresholds for CKD as shown in Fig. 3.

### Risk factors of kidney disease among study patients

Median age of 94 patients with kidney disease was 51 (IQR 35–67) years. Risk factors found to be associated with kidney disease were: age ≥ 60 years with OR 1.8 CI 0.98–3.31 *P* = 0.056, previous history of diarrhea or vomiting in past 3 months with OR 2.15 CI 1.33–3.47 *P* = 0.002 and heart failure with OR 3.52 CI 1.05–11.81 *P* =  < 0.042. Details are shown in Table 2 below. We used CKD EPI 2021 equation to determine KDIGO eGFR criteria of < 60 ml/min/1.73m^2^ in Table 2 below.

**Table 2:** Risk factors of kidney disease among study patients

	**Patients with eGFR of ≥ 60mls or proteinuria of trace or negative**	**Patients with** **eGFR < 60mls / min and or proteinuria ≥ + 1**			
**Statistic**	**Frequency (%)**	**Frequency (%)**	**Odds Ratio**	**95% CI**	***P*-value**
**Frequency (*N* = 357)**	263 (73.7)	94 (26.3)			
**Age: Median (IQR)**	45 (30—62)	51 (35—67)	1.01	1.00—1.02	0.069
**Age categories**					
18–35	93 (79.5)	24 (20.5)	1.00		
36–59	97 (72.9)	36 (27.1)	1.44	0.80—2.59	0.227
60 years and above	73 (68.2)	34 (31.8)	1.80	0.98—3.31	0.056
**Sex (%)**					
Female	124 (73.8)	44 (26.2)	1.00		
Male	139 (73.5)	50 (26.5)	1.01	0.63—1.63	0.955
**Alcohol Use**					
No	153 (72.5)	58 (27.5)	1.00		
Yes	110 (75.3)	36 (24.7)	0.86	0.53—1.40	0.551
**Previous or Current Cigarette smoking**					
No	214 (74.3)	74 (25.7)	1.00		
Yes	49 (71)	20 (29)	1.18	0.66—2.12	0.577
**HIV status**					
Negative	186 (73.5)	67 (26.5)	1.00		
Positive	77 (74)	27 (26)	0.97	0.58—1.64	0.919
**Had Diabetes mellitus**					
No	236 (75.2)	78 (24.8)	1.00		
Yes	27 (62.8)	16 (37.2)	1.79	0.92—3.50	0.087
**Had Hypertension**					
No	222 (77.9)	63 (22.1)	1.00		
Yes	41 (56.9)	31 (43.1)	2.66	1.55—4.59	< 0.001
**Previous febrile illness in past 3 months**					
No	117 (73.6)	42 (26.4)	1.00		
Yes	146 (73.7)	52 (26.3)	0.99	0.62—1.59	0.974
**Diarrhea or vomiting in past 3-months**					
No	177 (79.4)	46 (20.6)	1.00		
Yes	86 (64.2)	48 (35.8)	2.15	1.33—3.47	0.002
**Previous use of herbal remedies**					
No	235 (73.9)	83 (26.1)	1.00		
Yes	28 (71.8)	11 (28.2)	1.11	0.53—2.33	0.778
**Previous use of NSAIDS**					
No	243 (74.1)	85 (25.9)	1.00		
Yes	20 (69)	9 (31)	1.29	0.56—2.93	0.549
**Heart failure**					
No	258 (74.6)	88 (25.4)	1.00		
Yes	5 (45.5)	6 (54.5)	3.52	1.05—11.81	0.042

### Prevalence of chronic kidney disease at ≥ 90 days

#### Comparison of prevalence of CKD at ≥ 90 days determined by of proteinuria ≥ 1 + and or KDIGO eGFR criteria of < 60 ml/min/1.73m^2^ using various eGFR serum creatinine-based equations.

**Fig. 4:**
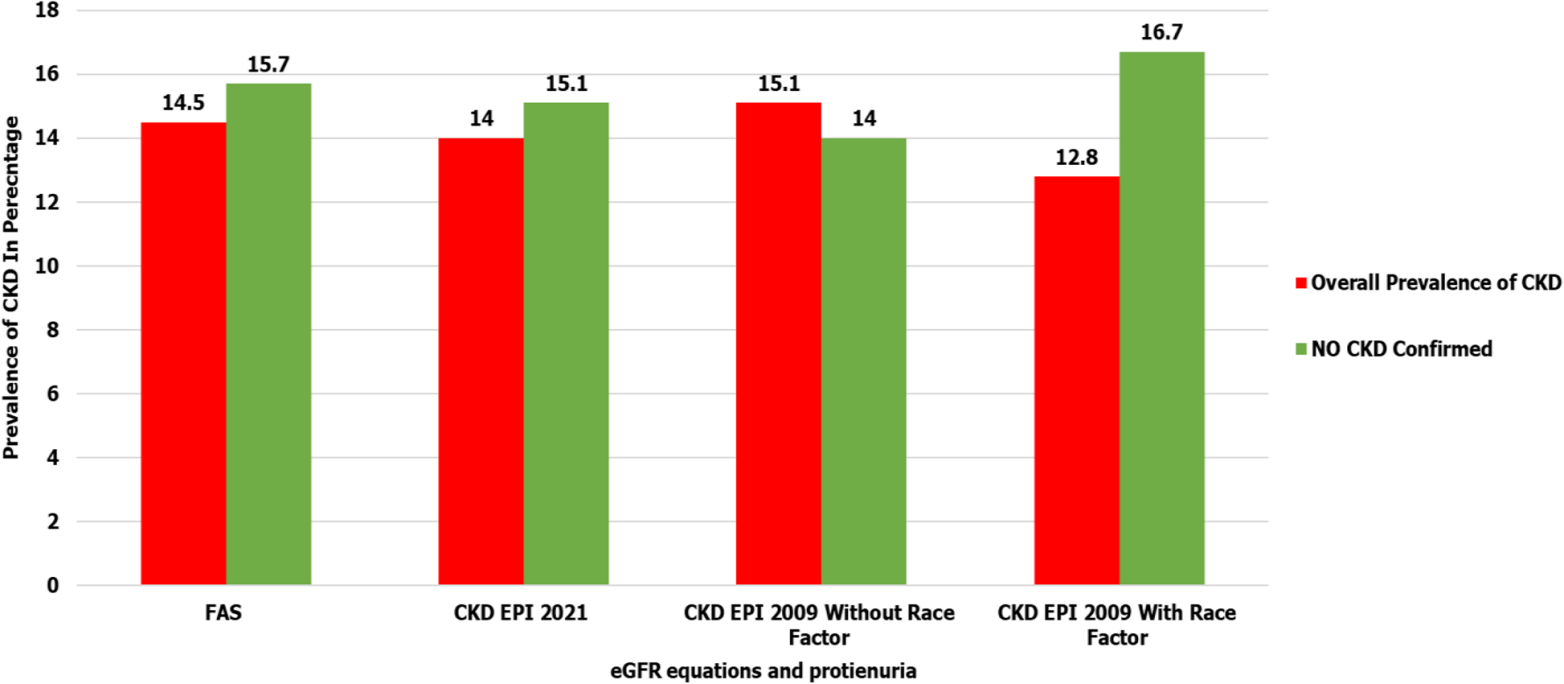
Comparison of prevalence of CKD at ≥ 90 days determined by of proteinuria ≥ 1 + and or KDIGO eGFR criteria of < 60 ml/min/1.73m^2^ using various eGFR serum creatinine-based equations. Bar chart shows that CKD confirmed by proteinuria ≥  + 1 and/or KDIGO eGFR criteria of < 60 ml/min/1.73m^2^ determined by CKD EPI 2009 without race factor identified the highest number of patients with CKD at 15.1% while CKD EPI 2009 with race factor identified the least number of patients at 12.8%

Out of 97 patients found to have kidney disease using proteinuria of ≥  + 1 and/or KDIGO eGFR criteria of < 60 ml/min/1.73m^2^ determined by CKD EPI 2009 without race factor equation, 11 patients were lost to follow-up, 61 died and confirmed CKD was highest at 13/86 (15.1%) in comparison with other eGFR equations. Details are shown in Fig. 4 below and in supplementary table 1 within additional file 2.

### Comparison of prevalence of CKD at ≥ 90 days while using KDIGO eGFR criteria of < 60mls/minute only and age adapted eGFR thresholds.

**Fig. 5:**
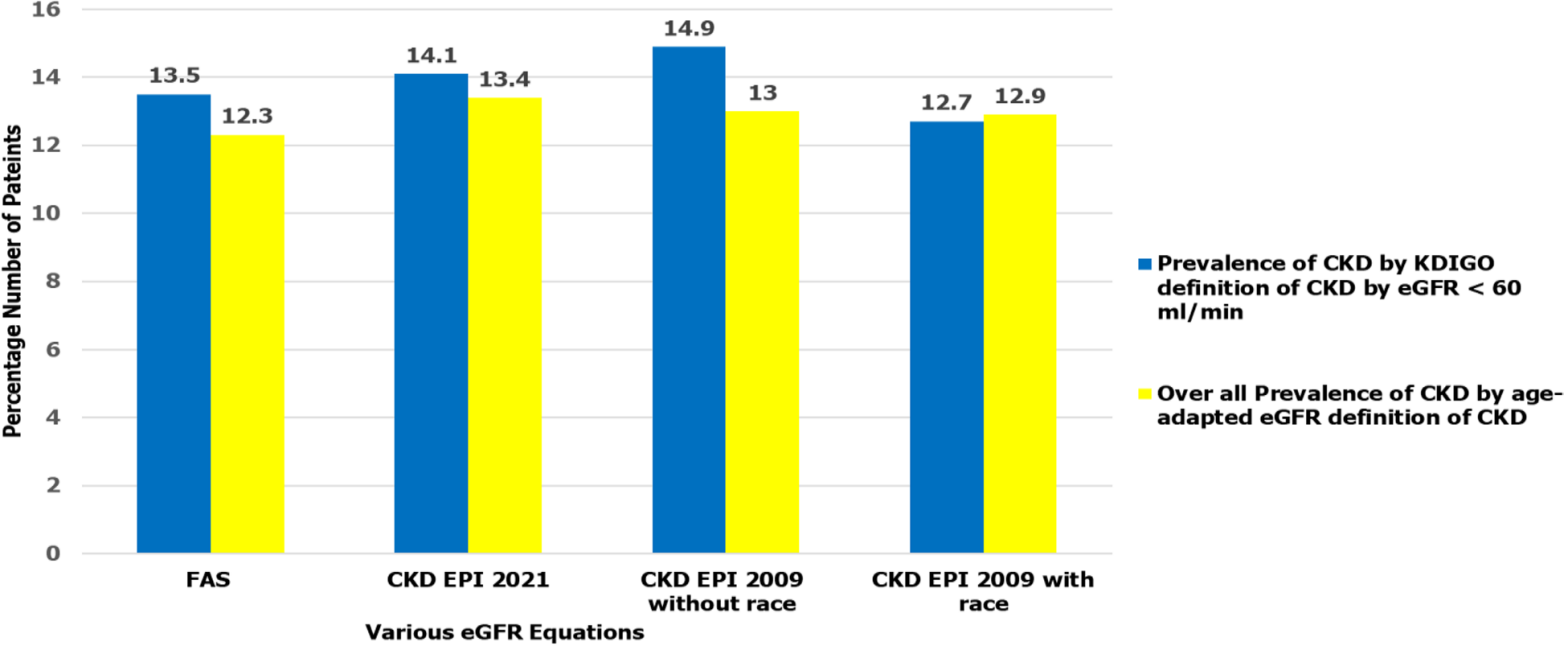
Comparison of prevalence of CKD after 90 days using KDIGO eGFR criteria of < 60mls/minute Vs age adapted eGFR thresholds to define CKD. Bar chart shows that KDIGO definition of CKD by eGFR < 60mls/minute/1.73m^2^ identifies slightly more patients with CKD than age adapted eGFR thresholds for CKD across most eGFR serum creatinine-based equations: FAS 13.5% vs 12.3%, CKD EPI 2021 14.1% vs 13.4%, CKD EPI 2009 without race 14.9% vs 13% respectively

Overall prevalence of confirmed CKD at 90 days by age-adapted eGFR threshold definition was compared with overall prevalence of confirmed CKD at 90 days by KDIGO eGFR criteria of < 60 ml/minute/1.73m^2^, details are shown in Fig. 5 below and supplementary table 2 within additional file 2.

## Discussion

As the overall burden of kidney disease increases, there is a need for increased screening, early identification and management of cases to prevent adverse outcomes from the disease processes. In this study, we compared prevalence of kidney disease determined by proteinuria of ≥  + 1 and or KDIGO eGFR criteria of < 60 ml/minute/1.73m^2^ using three creatinine-based equations among patients admitted on medical wards in Masaka RRH between September and November 2020. We found that at least two in every 10 patients had kidney disease with KDIGO eGFR criteria of < 60mls / minute /1.73m^2^ using full age spectrum and CKD-EPI 2009 without race factor equations and or proteinuria of ≥  + 1 showing the highest overall prevalence of kidney disease at 27.2%. These findings in our study are similar to those in a cross sectional study conducted in sub-Saharan African population which also found that adjustment for race did not improve the performance of GFR estimating equations and showed that creatinine-based FAS and CKD-EPI equations performed reasonably well [11]. Secondly our study revealed that KDIGO eGFR criteria of < 60mls/minute/1.73m^2^ for CKD identifies 0.3–3% slightly more patients with kidney disease than age adapted eGFR thresholds for CKD definition while using all eGFR serum creatinine-based equations. This is similar to findings of a nationwide study which was conducted in Iceland which revealed that using age-adapted eGFR thresholds resulted in a lower CKD prevalence [13].

Traditional risk factors found to be associated with kidney disease included: advanced age ≥ 60 years, hypertension, diabetes mellitus, heart failure, previous use of NSIADS and previous episodes of vomiting in past 3 months. The observed a high prevalence of kidney disease in this study could be due to multiple etiologies since patients were admitted on the medical wards due to various disease conditions. Many of the patients with kidney disease had known risk factors for kidney disease including advanced age ≥ 60 years, diabetes mellitus, previous episodes of vomiting in past 3 months, hypertension and heart failure [15–17].

Irrespective of the original cause of kidney disease, its high prevalence is of concern as this can easily progress to CKD, kidney failure and death in this population. Indeed, the observed prevalence is higher than what has been described previously in Uganda. A study by Nyende et al. in 2020 estimated the prevalence of kidney disease at 2.5% among HIV patients attending an out-patient clinic [18] and also in another study conducted in the general population which estimated the prevalence of abnormal eGFR at 19.8% [19]. The differences observed in our study estimate compared to these estimates could be mainly because of the differences in study setting or populations studied, varying definitions of kidney disease and use of different formulars to determine eGFR.

The incidence of CKD among patients with kidney disease was also high with one in every ten patients having CKD after 90-days of follow-up with CKD confirmed by proteinuria ≥  + 1 and/or KDIGO eGFR criteria of < 60 ml/min/1.73m^2^ determined by CKD EPI 2009 without race factor performing best with CKD incidence at 15.1% while CKD EPI 2009 with race factor performing worst with incidence of 12.8%. CKD was confirmed basing on evidence of ≥ 90 days of patients having KDIGO eGFR criteria of < 60 mls/minute/1.73m^2^ and or proteinuria of ≥ 1 + . This agrees with findings of a cross sectional study in sub-Saharan which found that the ethnic factor for CKD-EPI equations was not accurate in African population as it overestimates GFR. The lack of precision due to ethnic factor for the CKD EPI equations was probably because the factors were developed in African American people with different anthropometrical characteristics [11].

The study is not without limitations. Due to cost implications of diagnostic tests in our setting, we studied a relatively small number of patients and study patients were screened once (determination of SCr and performing urinalysis test) at the baseline screening of patients. However, screening patients twice would have provided more precise results. Secondly our study was conducted among admitted patients without comparison to healthy community populations.

## Conclusions

Prevalence of kidney disease was high. Use of KDIGO eGFR criteria of < 60mls / minute /1.73m^2^ using FAS and CKD-EPI 2009 without race equations identified more patients with CKD in comparison with other eGFR equations including the most recently introduced CKD EPI 2021. To promptly identity more patients with CKD, health care systems in sub-Saharan African countries should consider calculating eGFR using FAS or CKD-EPI 2009 without race factor equations during basic screening and management protocols.

## Supplementary Information

**Additional file 1.** Prevalence of abnormal kidney function and 90-days outcomes among patients admitted on medical wards of Masaka Regional Referral Hospital in Uganda.

**Additional file 2. Supplementary Table 1.** eGFR determined using different eGFR calculators and definitions of kidney disease. **Supplementary Table 2.** Shows prevalence of kidney disease at baseline and confirmed CKD at ≥ 90 days using age adapted eGFR threshold definitions by various eGFR calculators.

## Data Availability

All data generated or analyzed during this study are included in the supplementary additional file 1 and additional file 2.

## Abbreviations

CI: Confidence Interval
CKD: Chronic kidney disease
CKD-EPI: Chronic Kidney Disease Epidemiology Collaboration
eGFR: Estimated glomerular filtration rate
FAS: Full age spectrum Equation
KDIGO: Kidney Disease Improving Global Outcomes
IQR: Interquartile Range
RRH: Regional Referral Hospital
SCr: Serum Creatinine, OR: Odds Ratio
