# Supplementary material for: Comparison of the prevalence of kidney disease by proteinuria and decreased estimated glomerular filtration rate determined using three creatinine-based equations among patients admitted on medical wards of Masaka Regional Referral Hospital in Uganda: a prospective study
Source: BMC Nephrol. 2022 Jul 7;23:242. doi: 10.1186/s12882-022-02865-w (PMC9264612; doi:10.1186/s12882-022-02865-w)
Supplement: Supplementary file 2 — Additional file 2. Supplementary Table 1. eGFR determined using different eGFR calculators and definitions of kidney disease. Supplementary Table 2. Shows prevalence of kidney disease at baseline and confirmed CKD at ≥ 90 days using age adapted eGFR threshold definitions by various eGFR calculators. [file 12882_2022_2865_MOESM2_ESM.docx]

**Supplementary Table 1: eGFR determined using different eGFR calculators and definitions of kidney disease**

|  | **KDIGO definition** | | | |
| --- | --- | --- | --- | --- |
| **Number of patients=N (%)** | **FAS** | **CKD EPI 2021** | **CKD EPI 2009 without race** | **CKD EPI 2009 with race** |
| **Number of patients at baseline** | 357 | 357 | 357 | 357 |
| **Prevalence of decreased eGFR by KDIGO criteria of <60ml/min/1.73m² at baseline** | 81 (22.7) | 78 (21.9 | 81 (22.7) | 65(18.2) |
| **Prevalence of Kidney Disease at baseline (proteinuria ≥ +1 and or decreased eGFR of < 60mls/min by KDIGO)** | 97(27.2) | 94 (26.3) | 97 (27.2) | 82 (23) |
| **Number of patients lost to follow up** | 08 | 08 | 08 | 04 |
| **Patients who missed follow up due to initial study protocol for calculating eGFR using CKD EPI 2021 formula at baseline** | 06 | 00 | 03 | 00 |
| **Number of participants followed up at 90 days** | 83 | 86 | 86 | 78 |
| **Prevalence of CKD by decreased eGFR according to KDIGO criteria of <60ml/min/1.73m² at ≥ 90 days** | 10/74 (13.5) | 10/71 (14.1) | 11/74 (14.9) | 8/63 (12.7) |
| **Prevalence of CKD at ≥ 90 days (proteinuria ≥ +1 and or decreased eGFR by KDIGO criteria of < 60ml/min/1.73m²)** | 12/83 (14.5) | 12/86 (14) | 13/86 (15.1) | 10/78 (12.8) |

**N=number of patients, %=percentage**

**Supplementary Table 2: Shows prevalence of kidney disease at baseline and confirmed CKD at ≥ 90 days using age adapted eGFR threshold definitions by various eGFR calculators.**

|  | **Age-adapted definition** | | | |
| --- | --- | --- | --- | --- |
| **Number of patients =N (%)** | **FAS** | **CKD EPI 2021** | **CKD EPI 2009 without race** | **CKD EPI 2009 with race** |
| **Patients at baseline** | 357 | 357 | 357 | 357 |
| **Patients with eGFR <75 ml/min/1.73 m² < 40 years** | 25 | 24 | 26 | 24 |
| **Patients with eGFR <60 ml/min/1.73 m² between 40-65 years** | 32 | 35 | 35 | 29 |
| **Patients with eGFR <45 ml/min/1.73 m² for > 65 years** | 13 | 12 | 13 | 11 |
| **Overall prevalence of kidney disease at baseline by age-adapted eGFR threshold definition of CKD** | 70 (19.6) | 71 (19.9) | 74 (20.7) | 64 (17.9) |
| **Patients Lost to follow up by 90 days at follow up** | 05 | 04 | 05 | 02 |
| **Patients followed up at 90 days** | 65 | 67 | 69 | 62 |
| **Overall prevalence of confirmed CKD at ≥90 days by age-adapted eGFR threshold definition of CKD.** | 8/65 (12.3) | 9/67 (13.4) | 9/69 (13) | 8/62 (12.9) |

**N=number of patients, %=percentage**
